# Intimate partner violence, multiple mental health conditions and risk of small vulnerable newborn births: a maternity population-based data linkage study

**DOI:** 10.1016/j.eclinm.2026.103997

**Published:** 2026-05-29

**Authors:** Lisa Kent, Claire Kerr, Lorna Lawther, Kathryn M. Abel, Holly Hope, Krishnarajah Nirantharakumar, Kelly-Ann Eastwood, Aideen Maguire

**Affiliations:** a Administrative Data Research Centre Northern Ireland, Centre for Public Health, Queen's University Belfast, Belfast, UK; b School of Nursing and Midwifery, Queen's University Belfast, Belfast, UK; c Centre for Women's Mental Health, Division of Psychology and Mental Health, School of Health Sciences, Faculty of Biology Medicine & Health, The University of Manchester, Manchester, UK; d Greater Manchester Mental Health NHS Foundation Trust, Prestwich, Manchester, M25 3BL, UK; e Department of Applied Health Sciences, College of Medicine and Health, University of Birmingham, Edgbaston, Birmingham, UK; f Centre for Public Health, Queen's University Belfast, Belfast, UK; g Fetal Medicine Unit, St Michael's Hospital, University Hospitals Bristol and Weston NHS Foundation Trust, Bristol, UK

**Keywords:** Intimate partner violence, Domestic violence, Domestic abuse, Spouse abuse, Relationship abuse, Gender-based violence, Small vulnerable newborn, Premature birth, Infant, Low birth weight, Small for gestational age, Mental health

## Abstract

**Background:**

Small vulnerable newborn birth (SVN) encompasses infants born preterm, small for gestational age, or low birthweight, and accounts for most neonatal deaths worldwide. This maternity population-based administrative data-linkage cohort study aimed to estimate the risk of SVN associated with maternal intimate partner violence (IPV) experience.

**Methods:**

Records were included for women who accessed maternity services in Northern Ireland and had a singleton pregnancy, with liveborn or stillborn infants born 24–42 weeks’ gestation, with an estimated pregnancy start date 01 January 2011–31 December 2021. Pregnancy records were linked to community-dispensed prescriptions and hospital diagnoses data. Data were provided by the Honest Broker Service. IPV, maternal characteristics, and SVN were ascertained through maternity records. Mental health conditions were ascertained via medications or ICD-10 hospital diagnosis codes. Generalised estimating equations were utilised to perform modified Poisson regression with a log link, with clustering to account for women with more than one pregnancy during the study period. The risk of SVN given exposure to IPV was estimated, adjusted for number of mental health conditions and additional maternal characteristics.

**Findings:**

From 248,645 eligible pregnancies to 157,507 individual women, IPV was disclosed in 11,388 (4.6%) pregnancies (active: n = 3713, 1.5%; historical: n = 7675, 3.1%). The group with highest prevalence of IPV was pregnant adolescents (n = 995, 12.2%). Disparate prevalence estimates were also seen between the most (8.5%) and least (1.9%) deprived areas. Risk of SVN was increased for women reporting both active IPV (RR = 1.21, 95% CI: 1.13–1.31) and historical IPV (RR = 1.18 (95% CI: 1.12–1.25) independent of the number of coexisting mental health conditions and other maternal characteristics.

**Interpretation:**

IPV, both active and historical, is associated with an increased risk of birth of babies who are small and vulnerable, and so its detection, even IPV of historical nature, and response should be prioritised through training and creation of referral pathways.

**Funding:**

UKRI's ADRC NI (ES/W010240/1) & Strategic Priority Fund “Tackling multimorbidity at scale” programme (MR/W014432/1).

**Research in context**

**Evidence before this study**

A literature search was conducted on 19 March 2026 within MEDLINE only, using the following search strategy: (“intimate partner violence” OR “domestic violence” OR “domestic abuse” OR “spouse abuse” OR “relationship abuse” OR “gender-based violence”) AND (“pregnan$” OR “maternal” OR “small vulnerable newborn” OR “premature” OR “low birthweight” OR “small for gestational age”). Intimate partner violence (IPV) has been linked to a range of pregnancy outcomes, including preterm birth and infants born small for gestational age (SGA). Previous studies have examined single outcomes such as preterm birth or SGA in isolation however and have not utilised the unified concept of small vulnerable newborn (SVN) which encompasses preterm (<37 weeks), SGA (<10th centile of weight for gestational age), or low birthweight (<2500 g) infants, and accounts for most neonatal deaths worldwide. Previous study cohorts also tend to be restricted to subsets of the population and are mainly based in North America. Exposure to IPV has mostly been considered as a binary variable, which does not differentiate between historical and active IPV.

**Added value of this study**

Using a population-level maternity dataset linked to other routinely collected health and administrative data, this study provides evidence that IPV is associated with an increased risk of SVN. From 248,645 eligible pregnancies, 11,388 (4.6%) women disclosed IPV (active: n = 3713, 1.5%; historical: n = 7675, 3.1%). The group with highest prevalence of IPV were pregnant adolescents (n = 995, 12.2%). Disparate prevalence estimates were also seen between the most (8.5%) and least (1.9%) deprived areas. Both active IPV (RR = 1.21, 95% CI: 1.13–1.31) and historical IPV (RR = 1.18 (95% CI: 1.12–1.25) were associated with SVN, and these estimates were independent of co-existing mental health conditions and other important demographic, health-related, and socio-economic factors.

**Implications of all the available evidence**

SVN is a high prevalence-high impact outcome. Changes to maternity care policy and practice are required to ensure that IPV (past or present) is included as a risk factor for SVN so that appropriate care can be provided. Considering prevalence of IPV was greatest in pregnant adolescents, it is important that interventions to prevent IPV and support survivors begin early in the life course. The relationship between IPV and SVN is complex, and causation cannot be directly inferred. However, at a minimum, active or historical IPV disclosure is a useful marker for increased risk of SVN. There is a need for cross-sectoral policy to focus on IPV within preconception and interpregnancy health services and improve women's safe access to services. Conversely, pregnancy should also be considered a key risk period for targeted prevention of violence against women and girls.

## Introduction

Intimate partner violence (IPV) is both a criminal and public health problem^1^, ^2^, ^3^, ^4^ and a significant global concern, with the World Health Organization (WHO) estimating 20–33% prevalence across geographical regions.^1^ IPV includes threatening, controlling, coercive behaviour, violence or abuse (financial, physical, psychological/emotional, sexual) and can involve current or former intimate partners.^1^^,^^3^

The relationship between IPV and pregnancy is complex. Pregnancy is a critical timepoint, associated with initiation or escalation of IPV when it can be difficult for women to leave the relationship.^3^^,^^4^ Some forms of IPV, particularly pregnancy coercion and contraception sabotage, can result in pregnancy.^5^^,^^6^ IPV during pregnancy is implicated in maternal death^7^ and places women and children at increased risk of adverse outcomes, including hypertensive disorders of pregnancy, haemorrhage, miscarriage, preterm birth, small for gestational age (SGA), and low birthweight.^8^, ^9^, ^10^, ^11^, ^12^ However, there is as yet few robust estimates of the prevalence of IPV in pregnancy, with a previous systematic review limited to synthesising estimates based on small cohorts, often sampled from groups that are non-representative of populations, and with methodological deficiencies.^13^ Additionally, there are no population-based or survey-based studies of prevalence of IPV amongst pregnant women in Northern Ireland (NI), a context that may not reflect experiences of pregnant women in other nations. In NI, interview-based research has highlighted that experiences of IPV are shaped by a history of violent political conflict and conservative religious and cultural norms, and social attitudes continue to play a role despite the persistence of peace.^3^^,^^4^ This context has also been proposed to explain the relatively high prevalence of mental health conditions.^14^ Additionally, NI has the highest levels of deprivation compared to other UK nations,^15^ and together these factors result in a unique and complex context deserving of closer attention.

Most previous studies examining the relationship between maternal IPV exposure and infant outcomes explore single outcomes in isolation, are restricted to subsets of the population, do not differentiate between historical and active IPV, or are from North America. Examining outcomes such as preterm birth and SGA in isolation may underestimate poor outcomes, and hence the scale of impact of IPV. Small vulnerable newborn (SVN) is a unifying concept encompassing preterm (<37 weeks), SGA (<10th centile of weight for gestational age), or low birthweight (<2500 g) infants. SVN accounts for most neonatal deaths worldwide and is associated with life-long adversity.^16^ SVN infants experience immediate challenges with increased risks of morbidity and mortality. In childhood, adverse outcomes associated with SVN include growth and nutrition issues, developmental delay, neurodisability, neurodivergence, asthma, epilepsy, visual and hearing problems, and behavioural problems.^16^ Many of these problems continue into adolescence and adulthood, affecting the individual's physical and mental health as well as their educational attainment, employment prospects, and overall quality of life.^17^ Parents of SVN infants may also experience increased stress and decreased sleep quality.^18^^,^^19^ At a societal level, individuals born SVN have higher healthcare use, are less likely to contribute to human and economic capital and are more likely to die prematurely.^16^ It follows that SVN has substantial health, economic, and social population effects,^16^ and as such its surveillance at population level and further study of the relationship between IPV and SVN are justified to provide robust evidence of a need for multisectoral approaches to improve outcomes for women and children.

Maternal mental health conditions are also associated with both IPV exposure, and risk of preterm birth and SGA.^9^^,^^20^, ^21^, ^22^, ^23^ However, previous population-based studies of IPV in pregnancy do not account for maternal mental health.

Despite growing understanding of how IPV can affect women and children, it remains unclear how many pregnant women experience IPV, and the extent to which this influences outcomes in exposed women. This study aimed to address these evidence gaps and explore the prevalence of IPV disclosure in pregnancy in a population-level cohort, and estimate the association between active and historical IPV exposure and the composite outcome of SVN, specifically taking account of confounding by maternal mental health, and other relevant covariates. The objectives were to estimate (1) prevalence of active and historical IPV disclosure in women giving birth in an 11-year population-based cohort, (2) prevalence of indicators of poor mental health in women with and without IPV disclosure, (3) relative risks of SVN in women disclosing IPV, accounting for prior mental health indicators.

## Methods

### Data sources

Retrospective population, linked, de-identified, administrative health data were used to create the research dataset. The Health and Social Care Northern Ireland Business Service Organisation's (HSCNI BSO) Honest Broker Service (HBS), which is responsible for facilitating accredited researcher access to anonymised and pseudonomised health and social care data in NI, performed linkage between a number of datasets. Accredited researcher access was then facilitated within the HBS's Trusted Research Environment (United Kingdom Secure eResearch Platform, UK-SeRP), which is an accredited safe, secure and controlled environment for data linkage and analysis. The HBS also reviewed and approved any outputs from the project to ensure that “statistical disclosure control” was maintained; research outputs were only approved by HBS where counts of individuals were at least ten, thus reducing the re-identification risk and protecting the confidentiality of individuals. Pregnancy episodes to be included in the study cohort were ascertained from the Northern Ireland Regional Maternity Service Database (NIMATS), providing a “population spine”, to which all other datasets were then linked on an individual basis. Other administrative health datasets were linked to this population spine via individuals' unique Health and Care Number (HCN); hospital admission International Classification of Diseases 10th Revision (ICD-10) codes from The Patient Administration System (PAS) and community dispensed medications from The Enhanced Prescribing Database (EPD). There was complete HCN coverage in the study cohort within NIMATS, which enabled full linkage to PAS and EPD, where women had recorded interactions. Area level deprivation and settlement type as recorded in the NI Multiple Deprivation Measure (NIMDM)^24^ were also linked via postcode. All personal identifiers were removed from the data to ensure non-identifiability prior to researcher access. In addition, all outputs were screened by HBS staff before release to ensure confidentiality.

### Cohort selection–inclusion and exclusion criteria

All singleton pregnancies that resulted in live or stillborn infants born between 24 and 42-weeks’ gestation, with a pregnancy start date (estimated as 280 days before expected date of delivery) between 01 January 2011 and 31 December 2021 were considered for inclusion. Pregnancies were removed from the cohort if any of the following variables were missing: maternal age at birth (due to small numbers, postcode for linking to deprivation indices (due to small numbers), infant sex (required to calculate weight for gestational age), or infant birth weight (essential to outcome ascertainment). Births involving multiple foetuses were removed because of the increased likelihood of preterm birth or lower weight for gestational age (Figure S1).

### Exposures

IPV was ascertained for each recorded pregnancy using a NIMATS variable which records the woman's response to routine enquiry about either physical or emotional abuse.^25^ This routine enquiry variable is mandatory and is recorded during conversations between midwives and women that occur across many contacts during the antenatal, delivery and postnatal periods, when partners are not present. Due to the multiple timepoints when disclosure of IPV is discussed and captured in NIMATS, there are cases when multiple values are recorded within the same pregnancy and information may be discordant. When this was the case, the following hierarchy was utilised to deduplicate the variable for each pregnancy instance: “Disclosure—family receiving services” > “Disclosure—risk assessment and referral” > “Historical disclosure—no action requested: support and advice offered” > “no disclosure.” > “missing”. These levels were aggregated then as follows (1) any IPV: a binary variable which indicated if either current or historical IPV had been disclosed, and (2) timing of IPV: a categorical variable which indicated “Active IPV” regardless of whether services were being provided or referral required, “Historical IPV”, and “no disclosure/missing”. Overall, there were 2078 (0.84%) pregnancies where disclosure was missing throughout the entire pregnancy and due to small counts when stratified by maternal characteristics or SVN outcome, it was necessary to combine “missing” with “no disclosure” to protect confidentiality.

Pre-existing mental health conditions were initially identified using all three databases: PAS, EPD, and NIMATS. However, only mental health conditions indicated by medications in EPD or diagnoses in PAS contributed to the mental health variable in subsequent analyses. This approach is in line with methods detailed previously.^26^^,^^27^ Briefly, this involved identification of either primary or secondary ICD-10 diagnostic codes associated with hospital admissions within PAS, and British National Formulary codes and item names for community dispensed medications in EPD that were likely to indicate the presence of a condition of interest. For both, the look-back period was from inception of the database (PAS: January 2006; EPD: January 2010) until the estimated start date of pregnancy. Conditions were identified using a predefined list, codeveloped with people with lived experience, and included common mental health disorder: anxiety; depression; neurodevelopmental disorder; alcohol misuse; substance misuse; eating disorder; and “other”. Depression and anxiety counted as two separate conditions if diagnostic codes were available; if there were no diagnostic codes and only medications were detected, then common mental health disorder counted as one condition. A detailed list of conditions and the requirements for ascertainment are available in Table S1.

### Outcomes

In line with Ashorn and colleagues (2023),^16^ the outcome of SVN was ascertained from data contained in NIMATS and defined as an infant born at less than 37 weeks gestational age, small for gestational age (below tenth centile) or weighing less than 2500 g. Centile for weight-for-gestational-age was estimated from reference values based on a contemporary cohort of singleton births occurring in 2013–2014 in England and Wales, however ethnicity was not used in the calculation due to restrictions on researcher access to ethnicities other than white due small counts and the potential for disclosure.^28^

### Covariates

Variables that described women at the time of their first antenatal appointment were ascertained from NIMATS and NIMDM. Maternal age was categorised as <20 years, 20–24 years, 25–29 years, 30–34 years, 35–39 years, and ≥40 years unless statistical disclosure control dictated aggregated grouping, in which case maternal age was presented as <25 years, 25–34 years, and ≥35 years. Area level deprivation, as measured by overall NIMDM rank, was presented as either decile or quintile as statistical disclosure controls allowed. Settlement type was retained as bands A (Belfast City) to H (open countryside). Body mass index (BMI) was categorised as <18.5, 18.5–24.9, 25–29.9, 30–34.9, 35–39.9, ≥40 kg/m^2^, according to the WHO definition,^29^ or “missing”. Gravidity was categorised as 1, 2, 3, 4 and ≥ 5 pregnancies. Smoking status was categorised as currently smoking, previously smoked, non-smoking, and “missing”. Maternal age and gravidity were both fully complete in the study dataset. Ethnicity was grouped as “white”, “non-white” and “missing”, which has been the convention of administrative data research in NI, and deemed necessary due to the potential risk of disclosive small numbers within some ethnicities.

### Analysis

Prevalence of IPV disclosure in women who gave birth in NI maternity services was estimated for the full study population, and within subgroups stratified by maternal characteristics (age, area level deprivation, settlement type, ethnic group, BMI category, gravidity, pregnancy intention, smoking status, and alcohol consumption).

Prevalence of maternal mental health conditions was calculated for each IPV disclosure group (none, any, historical, and active) using medications (EPD) or diagnoses (PAS) as indicators. Prevalence of maternal mental health conditions recorded in NIMATS are provided separately in Table S2.

Generalised estimating equations were utilised to perform modified Poisson regression with a log link, with clustering to account for women with more than one pregnancy during the study period. This approach was used to estimate the relative risk of SVN in relation to (a) any IPV disclosure, and (b) timing of IPV- active or historical. Analyses were performed unadjusted, adjusted for covariates, and adjusted for covariates and number of mental health conditions (0, 1, ≥2 conditions).

Further a priori regression analysis was performed to explore the additive effect of coexisting IPV and mental health conditions. Comparison of the relative risk of SVN within intersecting IPV and maternal mental health condition(s) exposure groups was performed in reference to the group who had no IPV disclosure and no mental health conditions. The intersecting groups were as follows: no reported IPV and one mental health condition; no reported IPV and at least two mental health conditions; historical IPV and no mental health conditions; historical IPV and one mental health condition; historical IPV and at least two mental health conditions; active IPV and no mental health conditions; active IPV and one mental health condition; active IPV and at least two mental health conditions.

### Ethics approval and data governance

Ethical approval and consent to participate were not required for this study as it is limited to working with anonymised or pseudonymised data only within a Trusted Research Environment (TRE). This is in accordance with UK GDPR. This study was approved by The Health and Social Care Northern Ireland Honest Broker Service (HBS) Governance Board and Queen's University Belfast Research Governance and Ethics. This study is reported in accordance with the STROBE/RECORD guidelines for observational studies using routinely collected health data.

### Role of the funding source

The funders of the study had no role in study design, data collection, data analysis, data interpretation, writing of the report, or in the decision to submit the paper for publication.

## Results

### Prevalence of IPV disclosure in study cohort

From 248,645 singleton pregnancies to 157,507 individual women during the study period (2011–2021) in NI, IPV was reported in 11,388 (4.6%, 1 in 22) of pregnancies (Table 1). In 3713 (1.5%, 1 in 66) pregnancies, women were recorded as receiving support or in need of support during their pregnancy, referred to as “active IPV” within this study, and in 7675 (3.1%, 1 in 32) pregnancies, women reported historical IPV.

**Table 1:** Prevalence of pregnancies with a disclosure of IPV, stratified by timing of IPV and maternal characteristic.

	Full study cohort (N = 248,645 pregnancies)		No IPV disclosure (No/Not recorded) (n = 237,257 pregnancies, 95.42%)		IPV disclosure (Any) (n = 11,388 pregnancies, 4.58%)		IPV disclosure (Historical) (n = 7675 pregnancies, 3.09%)		IPV disclosure (Active) (n = 3713 pregnancies, 1.49%)	
	n	Column %	n	Row %	n	Row %	n	Row %	n	Row %
**Age at time of birth**										
<20 y	8153	3.3	7158	87.8	995	12.2	508	6.2	487	6.0
20–24 y	33,775	13.6	30,810	91.2	2965	8.8	1775	5.3	1190	3.5
25–29 y	67,464	27.1	64,236	95.2	3228	4.8	2185	3.2	1043	1.6
30–34 y	84,076	33.8	81,591	97.0	2485	3.0	1866	2.2	619	0.7
35–39 y	45,450	18.3	44,114	97.1	1336	2.9	1033	2.3	303	0.7
40 + y	9727	3.9	9348	96.1	379	3.9	308	3.2	71	0.7
**Multiple Deprivation Measure Decile**										
(Most deprived) 1	27,040	10.9	24,756	91.6	2284	8.5	1383	5.1	901	3.3
2	26,570	10.7	24,806	93.4	1764	6.6	1148	4.3	616	2.3
3	25,177	10.1	23,863	94.8	1314	5.2	870	3.5	444	1.8
4	27,936	11.2	26,628	95.3	1308	4.7	899	3.2	409	1.5
5	26,572	10.7	25,475	95.9	1097	4.1	767	2.9	330	1.2
6	24,570	9.9	23,616	96.1	954	3.9	688	2.8	266	1.1
7	25,878	10.4	24,977	96.5	901	3.5	607	2.4	294	1.1
8	23,798	9.6	23,040	96.8	758	3.2	543	2.3	215	0.9
9	21,577	8.7	20,942	97.1	635	2.9	479	2.2	156	0.7
(Least deprived) 10	19,527	7.9	19,154	98.1	373	1.9	291	1.5	82	0.4
**Settlement Type**										
(Belfast) A	37,328	15.0	34,828	93.3	2500	6.7	1651	4.4	849	2.3
(Derry) B	11,814	4.8	11,264	95.3	550	4.7	256	2.2	294	2.5
(Large towns) C	71,617	28.8	67,524	94.2	4093	5.7	2833	4.0	1260	1.8
(Medium towns) D	17,562	7.1	16,630	94.7	932	5.3	605	3.4	327	1.9
(Small towns) E	14,911	6.0	14,103	94.6	808	5.4	565	3.8	243	1.6
(Intermediate) F	10,402	4.2	10,047	96.6	355	3.4	236	2.3	119	1.1
(Villages) G	15,518	6.2	14,870	95.8	648	4.2	454	2.9	194	1.3
(Open countryside) H	69,493	28.0	67,991	97.8	1502	2.2	1075	1.6	427	0.6
**Ethnic group**										
White	211,191	84.9	200,865	95.1	10,326	4.9	6997	3.3	3329	1.6
Not white	7160	2.9	6827	95.4	333	4.7	189	2.6	144	2.0
Not known	30,294	12.2	29,565	97.6	729	2.4	489	1.6	240	0.8
**Body mass index**^a^										
<18.5 kg/m^2^	4622	1.9	4231	91.5	391	8.5	239	5.2	152	3.3
18.5–25 kg/m^2^	113,064	45.5	108,049	95.6	5015	4.4	3267	2.9	1748	1.6
25–30 kg/m^2^	73,524	29.6	70,446	95.8	3078	4.2	2102	2.9	976	1.3
30–35 kg/m^2^	32,897	13.2	31,293	95.1	1604	4.9	1144	3.5	460	1.4
35–40 kg/m^2^	13,905	5.6	13,149	94.6	756	5.4	534	3.8	222	1.6
≥40 kg/m^2^	6721	2.7	6323	94.1	398	5.9	305	4.5	93	1.4
Not recorded	3912	1.6	3766	96.3	146	3.7	84	2.2	62	1.6
**Gravida (number of pregnancies, including current)**^a^										
1	78,686	31.7	76,343	97.0	2343	3.0	1516	1.9	827	1.1
2	74,021	29.8	71,329	96.4	2692	3.6	1826	2.5	866	1.2
3	47,833	19.2	45,598	95.3	2235	4.7	1541	3.2	694	1.5
4	24,861	10.0	23,280	93.6	1581	6.4	1090	4.4	491	2.0
≥5	23,244	9.4	20,707	89.1	2537	10.9	1702	7.3	835	3.6
**Pregnancy intention**^a^										
Unplanned pregnancy	69,310	27.9	62,732	90.5	6578	9.5	4057	5.9	2521	3.6
Planned pregnancy	175,191	70.5	170,541	97.4	4650	2.7	3518	2.0	1132	0.7
Not recorded	4144	1.7	3984	96.1	160	3.9	100	2.4	60	1.5
**Smoking status**^a^										
Currently smoking	35,893	14.4	30,925	86.2	4968	13.8	3104	8.7	1864	5.2
Previously smoking	17,158	6.9	16,076	93.7	1082	6.3	794	4.6	288	1.7
Non-smoking	67,543	27.2	65,926	97.6	1617	2.4	1154	1.7	463	0.7
Not recorded	128,051	51.5	124,330	97.1	3721	2.9	2623	2.1	1098	0.9
**Alcohol consumption (any consumption ≥1 unit per week)**^a^										
Yes	1157	0.5	1050	90.8	107	9.3	66	5.7	41	3.5
No	247,488	99.5	236,207	95.4	11,281	4.6	7609	3.1	3672	1.5

a Recorded at booking/first antenatal appointment.

Within certain groups, the prevalence of IPV was higher: IPV was disclosed in 12.2% (1 in 8, n = 995) of pregnancies to adolescents (<20 years). Higher prevalence of IPV was reported in the most deprived areas (8.5%, n = 2284, 1 in 12 pregnancies), compared to the most affluent areas (1.9%, n = 373, 1 in 52 pregnancies). Generally, IPV was more frequently reported in pregnancies to women living in cities and towns than in more rural settlements. There were no meaningful differences between white (4.9%, n = 10,326) and non-white (4.7%, n = 333) ethnicities in our sample in the proportion of pregnancies with disclosure of IPV. Prevalence of IPV disclosure was also higher at extremes of maternal BMI. In pregnancies of women classed as underweight (BMI <18.5 kg/m^2^), prevalence of IPV disclosure was 8.5% (n = 391, 1 in 12), and in those with obesity class III (BMI ≥40 kg/m^2^) prevalence was 5.9% (n = 398, 1 in 17). Prevalence was higher in unplanned pregnancies (9.5%, n = 6578, 1 in 11) and those with gravidity of 5 or more (10.9%, n = 2537, 1 in 9). In pregnancies where women reported smoking or alcohol consumption during pregnancy, IPV disclosure was also higher–13.8% (n = 4968, 1 in 7) and 9.3% (n = 107, 1 in 11) respectively.

### Mental health conditions identified in pregnant women who disclosed IPV

Compared to pregnancies where there was no disclosure, pregnancies where women disclosed IPV showed almost twice the prevalence of having one or more mental health conditions (78.7%, n = 8958 versus 41.2%, n = 106,694), and seven times the prevalence of living with multiple mental health conditions (16.7%, n = 1900 versus 2.3%, n = 5540). This pattern persisted across all individual conditions (Table 2).

**Table 2:** Mental health conditions within the full study cohort, and within IPV exposure groups.

Conditions identified by medications (EPD) or secondary care ICD-10 diagnostic code (PAS)			Full study cohort (N = 248,645 pregnancies)		No IPV disclosure (No/Not recorded) (n = 237,257 pregnancies, 95.42%)		IPV disclosure (Any) (n = 11,388 pregnancies, 4.58%)		IPV disclosure (Historical) (n = 7675 pregnancies, 3.09%)		IPV disclosure (Active) (n = 3713 pregnancies, 1.49%)	
			n	%	n	%	n	%	n	%	n	%
At least 1 condition	EPD	PAS	106,694	42.9	97,736	41.2	8958	78.7	5976	77.9	2982	80.3
At least 2 conditions	EPD	PAS	7440	3.0	5540	2.3	1900	16.7	1150	15.0	750	20.2
Medication for CMHD	EPD		92,585	37.2	86,244	36.4	6341	55.7	4330	56.4	2011	54.2
Anxiety		PAS	1707	0.7	1334	0.6	373	3.3	224	2.9	149	4.0
Depression		PAS	3680	1.5	2809	1.2	871	7.7	561	7.3	310	8.4
Serious mental illness	EPD	PAS	9940	4.0	7998	3.4	1942	17.1	1197	15.6	745	20.1
Substance misuse	EPD	PAS	694	0.3	437	0.2	257	2.3	139	1.8	118	3.2
Alcohol misuse	EPD	PAS	1120	0.5	806	0.3	314	2.8	171	2.2	143	3.9
Neurodevelopmental disorder	EPD	PAS	588	0.2	439	0.2	149	1.3	90	1.2	59	1.6
Eating disorder		PAS	208	0.1	180	0.1	28	0.3	SDC	SDC	SDC	SDC
Other		PAS	6303	2.5	4719	2.0	1584	13.9	946	12.3	638	17.2

EPD, Enhanced Prescribing Database (community dispensed medications); PAS, Patient Administration System (ICD-10 diagnosis codes); CMHD, Common mental health disorder; SDC, Statistical disclosure control for counts <10 individuals.

Notes on conditions: CMHD includes cases where anxiolytics or antidepressants were identified but no diagnostic code detectable in PAS; anxiety includes diagnosis of phobia, panic disorder, or post-traumatic stress disorder; serious mental illness includes bipolar disorder, schizophrenia, affective psychosis, non-affective psychosis; neurodevelopmental disorder includes attention deficit hyperactivity disorder, autism, learning difficulties; “Other” includes obsessive compulsive disorder, personality disorder, dissociative disorder and self-harm (including suicide ideation).

When active IPV was disclosed, a higher prevalence of any mental health condition was observed compared to when historical IPV was disclosed (Table 2). In over 80% (n = 2982) of pregnancies with disclosed active IPV, women were found to have a pre-existing history recorded of at least one mental health condition, and over 20% (n = 750) had two or more pre-existing mental health conditions.

### Regression analysis: relative risk of SVN associated with IPV disclosure

The overall prevalence of SVN was 11.4% (n = 28,396) for the full cohort. Where IPV was disclosed, almost 1 in 5 (18.3%, n = 2086) babies were SVNs, compared to 1 in 9 (11.1%, n = 26,310) babies where IPV was not reported (Table 3). Pregnancies in women disclosing active IPV demonstrated a higher proportion again with 20.1% (n = 745) resulting in SVN. Pregnancies in women disclosing active IPV and living with multiple mental health conditions, showed the highest prevalence, with 25.9% (n = 194, 1 in 4), resulting in SVN.

**Table 3:** Number and percentage of small vulnerable newborn births across study groups.

Number and percentage of SVN in IPV disclosure groups				
Disclosure of IPV	Number of mental health conditions	Total number of singleton births (n = 248,645)	Small vulnerable newborns (n = 28,396, 11.42%)	
			n	Row %
No/not recorded	NA	236,987	26,310	11.09
Any IPV	NA	11,388	2086	18.32
Historical IPV	NA	7675	1341	17.47
Active IPV	NA	3713	745	20.06
**Number and percentage of SVN in IPV disclosure and mental health intersectional groups**				
No/not recorded	0	139,521	13,951	10.00
	1	92,196	11,286	12.24
	≥2	5540	1073	19.37
Any	0	2430	370	15.23
	1	7058	1257	17.81
	≥2	1900	459	24.16
Historical	0	1699	252	14.83
	1	4826	824	17.07
	≥2	1150	265	23.04
Active	0	731	118	16.14
	1	2232	433	19.40
	≥2	750	194	25.87

Footnote: NA, not applicable.

**Fig. 1:**
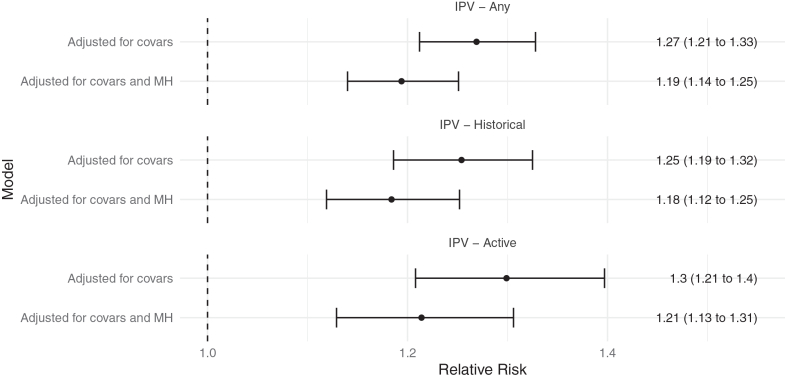
**Relative risk of SVN associated with IPV disclosure.** Relative risk of small vulnerable newborn in relation to disclosure of any IPV disclosure, historical IPV, active IPV, adjusted for covariates, and adjusted for covariates and number of mental health conditions (0, 1, ≥2 conditions).

After adjusting for covariates, pregnancies with disclosure of any IPV had a 27% greater risk of SVN than pregnancies where women did not disclose IPV (RR = 1.27, 95% CI: 1.21–1.33) (Fig. 1). When the number of mental health conditions was added to the model, the relative risk decreased, however the confidence intervals between the two results overlapped (RR: 1.19, 95% CI: 1.14–1.25).

Whilst active IPV conferred the greatest relative risk of SVN, the 95% confidence intervals overlapped for the results for active and historical IPV. After full adjustment for covariates and mental health, disclosure of active IPV still had a 20% higher risk of SVN compared to no disclosure of IPV (RR = 1.21, 95% CI: 1.13–1.31), whereas historical IPV conferred a relative risk of 1.18 (95% CI: 1.12–1.25).

Some categories within the covariates included in the fully adjusted models demonstrated statistically significant relationships with SVN: age ≥40 years compared to the reference group of 30–34 years (RR = 1.34, 95% CI: 1.27–1.42), first pregnancies (primigravida) compared to second pregnancies (RR = 1.46, 95% CI: 1.42–1.50), fifth or higher pregnancies (grand multigravida) compared to second pregnancies (RR = 1.11, 95% CI: 1.06–1.16), non-white ethnicity compared to white ethnicity (RR = 1.67, 95% CI: 1.57–1.76), underweight BMI compared to healthy BMI (RR = 1.58, 95% CI: 1.50–1.68), and currently smoking compared to non-smoking (RR = 2.03, 95% CI: 1.95–2.12). Compared to the most affluent decile, all other deciles demonstrated statistically significant increased risk of SVN ranging from 1.06 to 1.36. Broadly, deprivation deciles showed a dose–response type relationship with SVN, with the step increase in relative risk between subsequent deciles being greatest between decile 2, the second most deprived areas (RR = 1.26, 95% CI: 1.19–1.33) and decile 1, the most deprived areas (RR = 1.36, 95% CI: 1.28–1.44).

### Additional analysis: relative risk of SVN associated with coexisting IPV exposure and mental health condition(s)

In the fully adjusted regression models, the subgroups who had either active or historical IPV, in addition to two or more mental health conditions, demonstrated the highest relative risk of SVN birth- RR = 1.69 (95% CI: 1.48–1.94) and RR = 1.63 (95% CI: 1.45–1.83), respectively (Figure S2). Both active and historical IPV significantly increased the RR of SVN within subgroups who had no record of mental health conditions, and within subgroups who had one mental health condition.

When comparing IPV subgroups within those who had two or more mental health conditions, all groups had increased risk of SVN, with higher risk seen in the active and historical IPV groups, however, the 95% confidence intervals were wide and overlapped suggesting this difference was not significant.

Sensitivity analyses exploring risk of SVN by mental health status, adjusting for IPV show similar results [available on request].

## Discussion

This study found that IPV, both active and historical, is associated with an increased risk of birth of babies who are small and vulnerable. SVN occurred in a fifth of pregnancies where women disclosed any IPV, and this increased to a quarter of pregnancies where women disclosed active IPV and were also living with multiple mental health conditions. Pregnant women who disclosed IPV were more likely to be living with multiple mental health conditions, but an excess risk of SVN attributable to not only active IPV, but also historical IPV, remains after adjusting for mental health and other confounders.

Prevalence of IPV disclosure in pregnant women in NI (4.6% of pregnancies) was similar to general population estimates in the UK (4.8%).^30^ However, some groups had a higher prevalence–those who smoked during pregnancy (13.8%), adolescents aged under 20 years (12.2%), and those who had 5 or more pregnancies (10.9%). Whilst a previous report explored the impact of COVID-19 restrictions on monthly disclosure rates in pregnant women in NI,^31^ this is the first time that prevalence of IPV disclosure has been presented in this population. Prevalence of SVN in the presented study was 11.4%, and whilst it is difficult to make comparisons to other regions, a recent meta-analysis estimated global prevalence to be 11.7%.^32^ However, despite being comprehensive and robust, the cohorts in the meta-analysis only included live born infants, which may have resulted in an underestimation.

Comparison of the prevalence of IPV disclosure at the time of pregnancy to other regions in the UK, Ireland or Europe is not without limitations. A recent systematic review of global prevalence of IPV in pregnancy, provided highly dissimilar estimates for the only two European studies included, which were both based in Sweden (2.1% versus 24.2%).^33^ These differences perhaps reflect their different participant selection procedures, and support the justification for further population-based research of IPV disclosure in pregnancy.

Our findings add to our knowledge about maternal trauma, including historical IPV and its potential for intergenerational influence, however, comparison of findings of the association between IPV and SVN is difficult. Most studies included in two systematic reviews were conducted outside Europe, were not population-based, included only liveborn infants, or did not explore the composite outcome of SVN.^10^^,^^11^ Pooled results for “risk” attributable to IPV in both reviews ranged from 1.89 to 5.94 times higher for preterm birth, low birthweight, or SGA. Although analytic approaches are not comparable, this may indicate a stronger association between IPV and types of vulnerable newborn than observed in our study. However, our results are similar to the few population-based studies included in the reviews, suggesting that studies that used sampling or voluntary consent do not truly reflect population-level characteristics and risks, as volunteers and non-volunteers may differ systematically across sociodemographic characteristics.

Additionally, all deprivation deciles demonstrated increased risk of SVN compared to the most affluent decile, with risk increasing as deprivation increased, and the highest step increase observed between decile 2 (the second most deprived areas) and decile 1 (the most deprived areas). Risk within each decile of deprivation is not comparable to other nations, as NIMDM provides a relative measure within NI (i.e. areas are ranked according to absolute deprivation across multiple domains specific to NI). NI is a devolved nation within the UK, however, the relative nature of the NIMDM and its specificity to NI, in conjunction with other evidence that NI has higher levels of absolute deprivation compared with other UK nations, causes some difficulty in comparing prevalence and risk across similarly deprived areas across the UK.^15^

The NIMATS routine enquiry variable provided the opportunity to explore both active and historical IPV at population level, and complements other forms of prevalence estimates such as those obtained through survey-based research. The context lends itself to disclosure: conversations between midwives and women occur across many contacts during the antenatal, delivery and postnatal periods, with partners not present. Nevertheless, there is remaining potential for false negatives with estimates likely to underrepresent true prevalence as women experiencing IPV can have difficulties accessing antenatal services, and self-report of IPV may not be reflective of true prevalence.^34^^,^^35^ Further, we combined “missing” with “no disclosure” due to small counts and to protect anonymity of individuals in some sub-groups, but these values may not represent the same situation: “missing” may indicate the partner was present, preventing enquiry.

The ability to link additional datasets allowed us to ascertain covariates across biological, psychological, and socioeconomic domains, providing robust estimates of the risk of SVN attributable to IPV. Other studies of risk factors in pregnancy tend to focus on exposures within a single domain.^36^ It must be acknowledged, however, that there may be collinearity amongst some of these variables, and thus some estimates related to covariates may be imprecise.

As with most administrative data research in NI to date, in this study ethnicity was grouped “white”, “non-white”, and “unknown” because of potential disclosive small numbers within some ethnicities. This prevented detailed exploration of differences between ethnic groups, and for ethnicity to be considered when calculating weight-for-gestational-age centiles. Future work in NI should explore approaches that balance detailed analysis of ethnicity differences with protecting confidentiality.

A further limitation is the missingness in the smoking variable used within this study. With over 50% missingness in smoking status, results associated with this should be interpreted with caution. In addition, it is acknowledged that utilising medications as a proxy for mental health conditions has the potential to miss individuals who are accessing non-pharmacological treatments or not accessing treatment at all, which may be more common in the period before a planned pregnancy. However, utilisation of a full look-back period from the inception of the EPD facilitates identification of any history of use of psychotropic medications prior to pregnancy. Conversely, utilisation of certain psychotropic medications may miss-identify individuals as having a mental health condition, for example, those who are receiving antidepressant medications for other indications. However, the validity of this method for identifying population-level patterns in mental health conditions has been demonstrated against self-reported Census data^37^

Prevention of SVN births has typically focused on medical interventions.^38^ Our findings suggest that IPV is a significant and high prevalence risk factor that needs a broader approach to improve outcomes for women and their babies, and a need for early prevention with a focus on the highest risk groups.

Population-level screening for IPV during antenatal care must now be linked to other key elements in women's lives implicated by our findings such as smoking status, maternal age, and gravidity. Routine enquiry provides a key opportunity to explore historical IPV, consider the prospective effects on the pregnancy and implement mitigating care.^39^ Guidance specific to midwives in NI sets out how to enquire, recognise and respond to IPV, however, they are oriented to tackling active IPV and focus mainly on personal safety.^25^ Whilst there is no doubt that this is important, this study also suggests that there is a gap in guidance for reducing health related risks in both women and infants associated with historical IPV. It is important that women who have lived-experience of IPV are involved in service development and implementation. However, as a minimum, training antenatal staff in trauma-informed care might mitigate some of the effects of IPV.^40^ Changes to maternity care policy and practice are also required to ensure that IPV (past or present) is included as a risk factor for SVN so that appropriate care can be provided.

Our study has demonstrated that IPV disclosure is greater in women with poor preconception and interpregnancy health indicators, including smoking, underweight, and pregnancy planning. This may in part be due to experience of IPV in the form of sexual assault or reproductive coercion which limits the ability to plan or prepare for pregnancy, or coercive control limiting access to health services. At the least, preconception and interpregnancy care and reproductive and sexual health services could be improved by embedding IPV prevention and support, particularly for younger women and women with mental health conditions. A scoping review of guidelines in North America and Australia/Oceania found that preconception IPV screening was recommended, however there is scant detail on recommendations for responding to IPV.^41^ Additionally, a further scoping review based in the UK and Ireland found that, at present, IPV is not a prominent factor in preconception and interpregnancy health polices, strategies, and guidelines.^42^ Additionally, health system design that reflects women's difficulties in accessing these services is needed, particularly in the context of coercive control.^43^ An example of efforts in the NI context to recognise and respond to IPV around the time of pregnancy has recently been evaluated within the “Family Nurse Partnership”, which is an evidence-based prevention program, for younger women preparing to parent for the first time and living in social and economic disadvantage.^44^ The current study suggests that support is also required not just for first-time parents, but also for those who have had a higher number of pregnancies, as these were some of the groups with the highest prevalence of IPV, both active and historical. A recent scoping review has highlighted the need to evaluate efforts to reduce IPV and treat the adverse effects of a history of violence,^45^ and there may be scope to do so using population-based routinely collected health data such as was used in the presented study.

This study highlights the importance of historical IPV on pregnancy outcomes providing new insight into the legacy of trauma. Risk remained increased even after adjusting for mental ill-health, which suggests that there may be additional related factors, unmeasured in this study. There may be additional social, structural, or physical health factors to explore, and further research may also be indicated to explore potential neurobiological effects of stress imprinting on the next generation, and uncover why historical IPV disclosure remains important in SVN births.

Longstanding political focus on reducing violence against women across nations has led to little change. However, as acknowledged in recently published strategies for tackling violence against women and girls, both in NI and across other UK nations, better recording is an important first step to wider recognition and action.^46^, ^47^, ^48^, ^49^, ^50^, ^51^ The reproductive period is a key risk period, and also a time when women have many routine health contacts providing ample opportunity for intervention. Therefore, the current lack of focus on preconception, pregnancy, and postnatal risk periods needs to be re-considered across policy and service provisions in NI's Programme for Government and associated strategies and delivery plans for tackling violence against women and girls.^46^, ^47^, ^48^ Preventing IPV and supporting victims may represent upstream potential to prevent SVN births, with opportunities to intervene across health and social care, statutory agencies, and community groups. Our findings provide a reliable, contemporary, population-based benchmark against which to measure the impact of future policy and interventions aimed at prevention of IPV and amelioration of its impact.

Victims under 16 years, likely to be legally considered child abuse rather than IPV, are often missing from national IPV statistics.^52^ However, our study reports the highest prevalence of IPV in pregnant adolescents. This gap in reporting may have important consequences. Closer attention in national statistics and surveillance systems to younger pregnant women, who are also most likely to develop postnatal mental health conditions,^53^ may enable better understanding of the evolution of IPV, reproductive outcomes, and mental health across the lifespan, and identify opportunities to intervene early to protect women, their families, and future generations.

In conclusion, intimate partner violence, current and historical, is associated with an increased risk of babies who are born small and vulnerable. The high prevalence of IPV disclosure in a contemporary population of pregnant women in the UK, particularly younger women, is concerning. Pregnancy is a key time to monitor prevalence of IPV, identify women with current or historical exposure, and act to mitigate the risks to women and babies.

## Contributors

LK, KMA, HH, KN, KAE and AM contributed to the conception of this work. LK, KAE and AM contributed to the planning of this work. LK contributed to carrying out and analysing this work. LK and AM had direct access to and verified the underlying data reported in the manuscript. LK, CK, LL and AM contributed to draughting this work. All authors (LK, CK, LL, KMA, HH, KN, KAE, AM) contributed to interpretation and substantial revision of this work. All authors (LK, CK, LL, KMA, HH KN, KAE, AM) approved the submitted version and accept responsibility for the paper as published.

## Data sharing statement

The individual-level data that support the findings of this study are available from Honest Broker Service (HBS) within the Business Services Organisation Northern Ireland (BSO), but restrictions apply to the availability of these data, which were used under licence for the current study, and so are not publicly available. For further details and meta-data relating to the databases used in this study, please contact HonestBrokerService@hscni.net or refer to the Honest Broker Service website available at https://bso.hscni.net/directorates/digital/honest-broker-service/honest-broker-service-researcher-access/metadata/.

## Declaration of interests

LK, CK, LL, HH, KA, KAE and AM declare that they have no interests to disclose. KN declares grants from NIHR, UKRI/MRC, Kennedy Trust for Rheumatology Research, Health Data Research UK, Wellcome Trust, European Regional Development Fund, Institute for Global Innovation, Boehringer Ingelheim, Action Against Macular Degeneration Charity, Midlands Neuroscience Teaching and Development Funds, South Asian Health Foundation, Vifor Pharma, College of Police, and CSL Behring. KN declares consulting fees from BI, Sanofi, CEGEDIM, Dexter AI and MSD. KN declares a leadership/fiduciary role NICST, a charity and OpenClinical, a Social Enterprise and Dexter.
