# Supplementary material for: Intimate partner violence, multiple mental health conditions and risk of small vulnerable newborn births: a maternity population-based data linkage study
Source: eClinicalMedicine. 2026 May 29;96:103997. doi: 10.1016/j.eclinm.2026.103997 (PMC13240769; doi:10.1016/j.eclinm.2026.103997)
Supplement: Table S1 [file mmc1.docx]

| Table S1: Requirements for identifying mental health conditions in Enhanced Prescribing Database (EPD) and the Patient Administration System (PAS) | | | |
| --- | --- | --- | --- |
| Condition | Notes | Database | Requirements |
| Medication for common mental health disorders | Where cases did not have evidence of ICD-10 codes for anxiety or depression, but medications were recorded. | EPD | BNF 4.3 (Antidepressant drugs, excluding amitriptyline)  BNF 4.1.2 (Anxiolytics)  Propranolol 10mg or 40mg |
| Anxiety | Including phobia, panic disorder, post traumatic stress disorder. | PAS | ICD-10 (3 character): F40, F41  ICD-10 (4 character): F43.1 |
| Depression | - | PAS | ICD-10 (3 character): F32, F33 |
| Serious mental illness | Including bipolar disorder, schizophrenia, affective psychosis, non-affective psychosis. | EPD | (2 Group A prescriptions within any 12 months AND any lifetime group B/C prescriptions) **OR (**2 group B prescriptions within any 12 months) **OR** (2 group C prescriptions within any 12 months)  **Group A:**  BNF 4.3 (Antidepressant drugs, excluding amitriptyline)  BNF 4.1.2 (Anxiolytics)  **Group B:**  BNF 4.2.1 (Antipsychotic drugs, excluding prochlorperazine)  BNF 4.2.2 (Antipsychotic depot injections)  **Group C:**  BNF 4.2.3 (Drugs used for mania and hypomania: lithium, asenapine) |
|  |  | PAS | ICD-10 (3 character): F20, F21, F22, F25, F28, F29, F30, F31 |
| Substance misuse |  | EPD | Methadone (identified by name, not BNF) |
|  |  | PAS | ICD-10 (3 character): F11, F12, F13, F14, F15, F16, F18, F19 |
| Alcohol misuse |  | EPD | BNF 4.10.1 (alcohol dependence: acamprosate, disulfiram, nalmefene) |
|  |  | PAS | ICD-10 (4 character): F10.1, F10.2, F10.3, F10.4, F10.5, F10.6, F10.7, F10.8, F10.9, E24.4, G31.2, G62.1, G72.1, K29.2, K85.2, K86.0, Z50.2, Z71.4 |
| Neurodevelopmental disorder | Including attention deficit hyperactivity disorder, autism, learning difficulties. | EPD | Methylphenidate (identified by name, not BNF) |
|  |  | PAS | ICD-10 (3 character): F90, Q90, F70, F71, F72, F73, F78, F79  ICD-10 (4 character): F84.0, F84.1, F84.5, F81.9 |
| Eating disorder |  | PAS | ICD-10 (4 character): F50.0, F50.1, F50.2, F50.3 |
| Other | Includes obsessive compulsive disorder, personality disorder, dissociative disorder, self-harm (including self-harm and suicide ideation) | PAS | ICD-10 (3 character): F42, F60, F61, F44, X60, X61, X62, X63, X64, X65, X66, X67, X68, X69, X70, X71, X72, X73, X74, X75, X76, X77, X78, X79, X80, X81, X82, X83, X84  ICD-10 (4 character): Y87.0, Z91.5 |

EPD (Enhanced Prescribing Database): At least 2 instances of medications being dispensed within any 12-month period prior to the estimated start date of pregnancy

PAS (Patient Administration System): At least one ICD-10 code recorded as primary or secondary diagnosis, associated with any admission to secondary care hospital prior to the estimated start date of pregnancy
