# Supplementary material for: Intimate partner violence, multiple mental health conditions and risk of small vulnerable newborn births: a maternity population-based data linkage study
Source: eClinicalMedicine. 2026 May 29;96:103997. doi: 10.1016/j.eclinm.2026.103997 (PMC13240769; doi:10.1016/j.eclinm.2026.103997)
Supplement: Table S2 [file mmc2.docx]

| **Table S2: Mental health conditions identified in the Northern Ireland Maternity System (NIMATS) within the full study cohort, and within IPV exposure groups** | | | | | | | | | | |
| --- | --- | --- | --- | --- | --- | --- | --- | --- | --- | --- |
|  | **Full cohort** | | **No IPV** | | **IPV - any** | | **IPV - Historical** | | **IPV - Active** | |
|  | **n** | **%** | **n** | **%** | **n** | **%** | **n** | **%** | **n** | **%** |
| **Identified through maternity records** | | | | | | | | | | |
| At least 1 MH | 69,398 | 27.91 | 61,453 | 25.90 | 7,945 | 69.77 | 5,345 | 69.64 | 2,600 | 70.02 |
| At least 2 MH | 12,927 | 5.20 | 10,017 | 4.22 | 2,910 | 25.55 | 1,901 | 24.77 | 1,009 | 27.17 |
| At least 1 serious MH* | 35,078 | 14.11 | 29,826 | 12.57 | 5,252 | 46.12 | 3,477 | 45.30 | 1,775 | 47.81 |
| Schizophrenia* | 131 | 0.05 | 87 | 0.04 | 44 | 0.39 | 23 | 0.30 | 21 | 0.57 |
| Bipolar affective disorder* | 612 | 0.25 | 427 | 0.18 | 185 | 1.62 | 107 | 1.39 | 78 | 2.10 |
| Postnatal psychosis* | 579 | 0.23 | 464 | 0.20 | 115 | 1.01 | 71 | 0.93 | 44 | 1.19 |
| Severe depression* | 15,789 | 6.35 | 12,694 | 5.35 | 3,095 | 27.18 | 2,014 | 26.24 | 1,081 | 29.11 |
| Severe eating disorder* | 1,590 | 0.64 | 1,318 | 0.56 | 272 | 2.39 | 191 | 2.49 | 81 | 2.18 |
| Severe OCD* | 835 | 0.34 | 684 | 0.29 | 151 | 1.33 | 101 | 1.32 | 50 | 1.35 |
| Other serious MH disorder* | 17,870 | 7.19 | 15,808 | 6.66 | 2,062 | 18.11 | 1,402 | 18.27 | 660 | 17.78 |
| At least 1 minor MH** | 42,852 | 17.23 | 37,986 | 16.01 | 4,866 | 42.73 | 3,256 | 42.42 | 1,610 | 43.36 |
| Anxiety/panic attacks** | 27,231 | 10.95 | 23,864 | 10.06 | 3,367 | 29.57 | 2,263 | 29.49 | 1,104 | 29.73 |
| Mild/moderate/postnatal depression** | 18,576 | 7.47 | 16,540 | 6.97 | 2,036 | 17.88 | 1,346 | 17.53 | 690 | 18.58 |
| Minor eating disorder** | 884 | 0.36 | 748 | 0.32 | 136 | 1.19 | 89 | 1.16 | 47 | 1.27 |

*Obtained from NIMATS variable “PMH_MENTAL_HEALTH”. **Obtained from NIMATS variable “PMH_MH_MINOR” .
