# Supplementary figures and images for: Intimate partner violence, multiple mental health conditions and risk of small vulnerable newborn births: a maternity population-based data linkage study
Source: eClinicalMedicine. 2026 May 29;96:103997. doi: 10.1016/j.eclinm.2026.103997 (PMC13240769; doi:10.1016/j.eclinm.2026.103997)

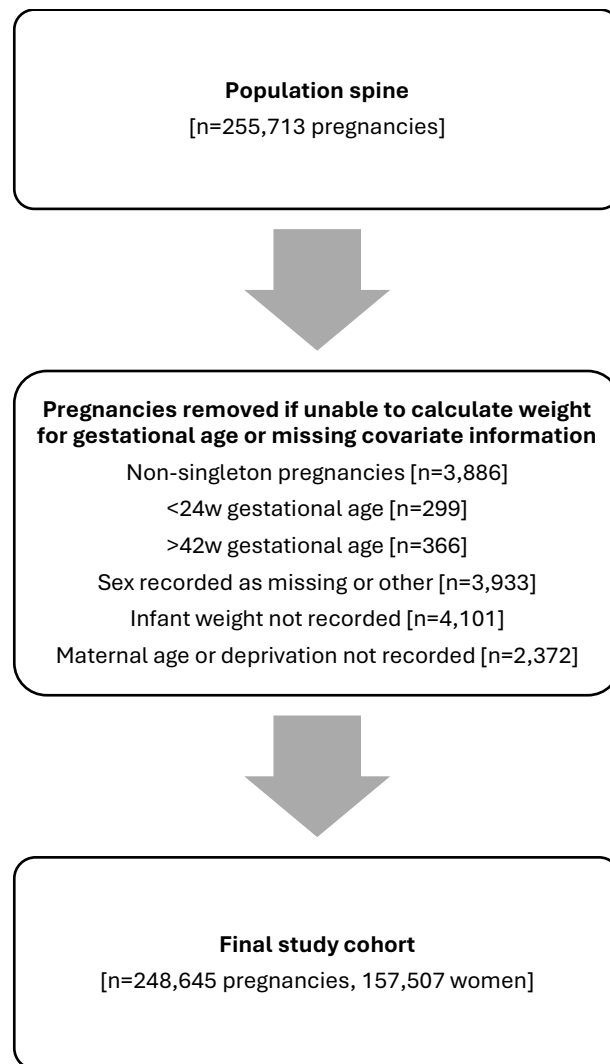

**Figure S1: Flow diagram: Study cohort selection**

Supplement: Figure S1 [file mmc3.pdf]
