# Supplementary material for: Intimate partner violence, multiple mental health conditions and risk of small vulnerable newborn births: a maternity population-based data linkage study
Source: eClinicalMedicine. 2026 May 29;96:103997. doi: 10.1016/j.eclinm.2026.103997 (PMC13240769; doi:10.1016/j.eclinm.2026.103997)
Supplement: Figure S2 [file mmc4.pdf]

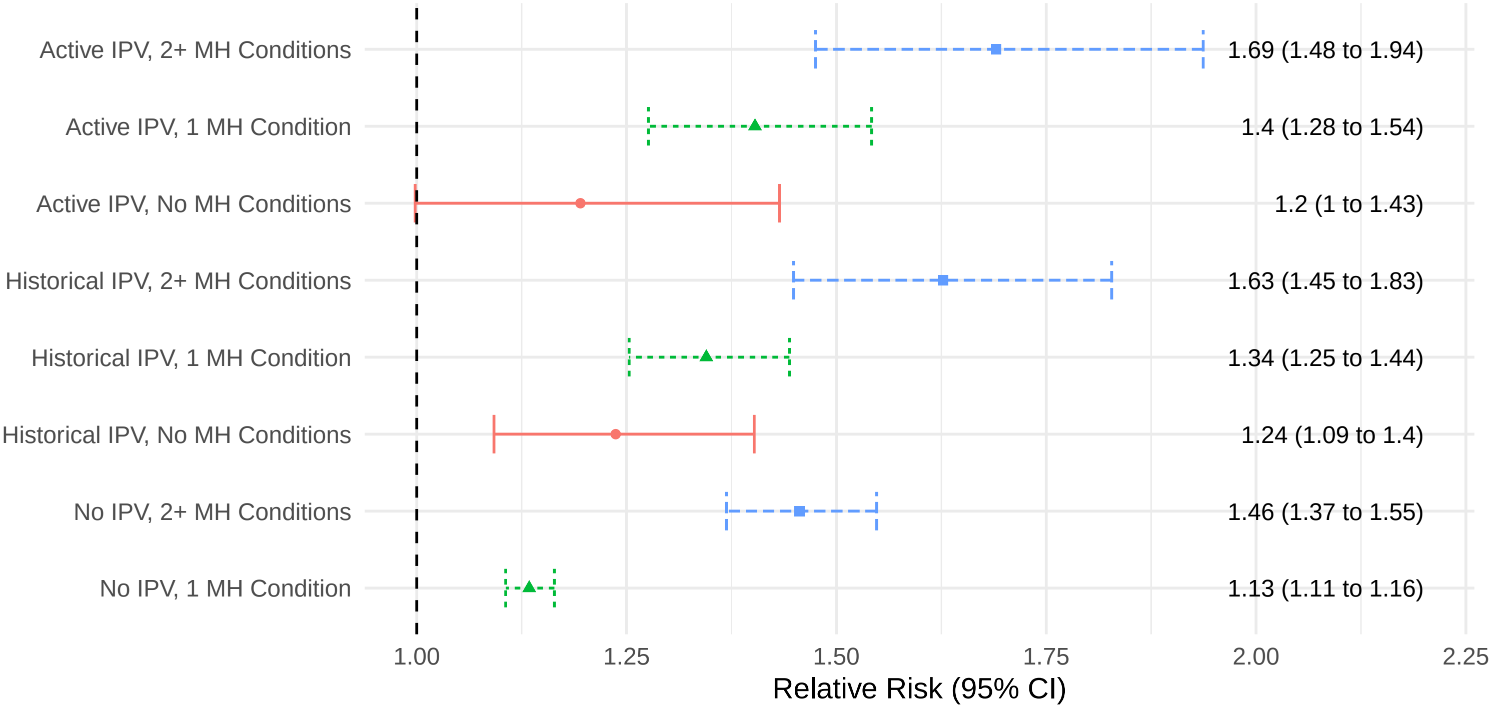

Figure S2: Relative risk of SVN associated with coexisting IPV exposure and mental health condition(s)
